# A Treatise on Etherization in Child-Birth

**Published:** 1848-12

**Authors:** 


*A Treatise on Etherization in Child-Birth. Illustrated by five hundred and sixty-one cases.* By WALTER CHANNING, M.D., Professor of Midwifery and Medical Jurisprudence in the University of Cambridge. 8vo. pp. 400. William D. Ticknor and Company: Boston, 1848.

Professor Channing was one of the first, if not the very first, to employ ether for the purpose of relieving the pains of child-birth; it is peculiarly appropriate, therefore, that such a work as the present should come from him, as well as from the city to which we are indebted for our knowledge of the wonderful powers of the agent. His position, and great experience, too, with its use, imposed this obligation upon him, and we are sure that every member of the profession, who has the opportunity of reading the work, will unite with us in returning thanks to him for the labour and ability he has bestowed upon it. The abundant facts contained in the volume, if they do not establish the propriety of using ether for the relief of ordinary labour pains, at least justify, beyond cavil, we think, the utility of the practice in cases of extreme suffering, where there is no extraordinary mechanical difficulty. We confess to having always taught and practised upon the doctrine, that "*meddlesome midwifery is bad,*" and we are not at all prepared to abandon the precept. While, therefore, we cannot *yet* go the length of the author, either in his practice or his reasoning on this subject, we are ready to yield,—have yielded, in fact,—much of our apprehension of danger from the use of ether, upon the ground, that we hope always to be ready to surrender preconceived notions to extensive and enlightened experience. If we had seen nothing of the effects of the remedy ourselves, we should feel bound to yield our prejudices to the authority of facts so numerous, observed by such men as Simpson, Channing, and many others of acknowledged competency, who have reported upon the subject. Having premised thus much, we proceed to notice some of the most prominent points discussed by our author.

Professor Channing contends, that pain is not an essential condition of the parturient process. "The *functional* department of labour," he remarks, "is the *contraction* of the womb, the dilatation of its mouth, vagina, and external organs, which are no more necessarily painful than are those which carry forward, and expel the contents of the rectum or bladder. There is no pain in the purely functional action of the uterus. Pain is the consequence of *resistance* to the contractions of the womb, which the moving body, the fœtus, encounters in its progress to birth."

This is a most important position, and if it shall be established as sound physiological doctrine, a great objection to the general employment of anæsthetic agents in midwifery will be removed. The only question to be determined then will be, whether we are possessed of means for preventing pain, without interfering with the proper progress of labour, and without additional risk to the mother or offspring. Our author maintains the affirmative.

The following is his explanation of the manner in which etherization acts in obviating the perils of labour: "It gives the demanded relief, by increasing dilatability, diminishing or suspending sensibility, preventing exhaustion, increasing the secretions, taking away the disturbing action of the will; and thus produces results which strike the observer of the first case in which he witnesses it, as if a miracle had been performed in his presence." Such results would appear miraculous indeed. But is the picture not overdrawn? Has imagination lent no colouring to it? It does seem to our cold and skeptical eyes, that enthusiasm—a generous and noble enthusiasm, we grant—has had a full share in the delineation. That etherization diminishes or suspends sensibility to a great degree during labour, and takes away "the disturbing action of the will," accords with our own observation; but that it increases the secretions, and also the dilatability, without suspending uterine contraction, experience has not taught us, neither can we understand how it should be so.

"Let it then be distinctly borne in mind," says our author, "that etherization is not used to suspend *uterine contractions*, (which it most rarely does,) but to prevent *pain*, and, in this way, to make labour safe and happy to both mother and child, and to secure a successful convalescence." He declares, "that in not a single instance of the thousands of recorded cases of child-birth has there been a single untoward result met with during etherization; and what further argument," he asks, "do we want to support the position, that this agency in painful labour, is not only most reasonably demanded by the sufferer, but that it is the solemn duty of the profession to afford to such suffering its certain relief?"

This assertion is hardly reconcilable with the following observation in the concluding part of the same introductory chapter "It is no part of the purpose of the following treatise to teach, or to leave it to be inferred, that no untoward results have not [?] followed, or will not again follow etherization. But I can and do say, that I have not met with an untoward result in any case of midwifery in which etherization has been induced, which, by any violence or ingenuity of explication, can be ascribed to this state as its cause. I have met with no record of such."

Perhaps some of the author's readers may think, in regard to this expression of his belief, that others might arrive at a different conclusion from a consideration of the same facts. While it cannot be denied, that Dr. Channing has been eminently *fortunate* in his use of ether during labour, it is difficult to divest oneself of the impression, that his enthusiasm in a most interesting subject has, imperceptibly to himself, occasionally influenced his perception of things. We do not charge this upon him; but it is difficult for any one to write with so much zeal, on *any subject*, without being obnoxious to it.

The following remarks are quoted from the writings of our illustrious townsman, the late Professor B. Rush. They are certainly very remarkable, and may almost be regarded as prophetic.

"By some divines, these symptoms, and particularly pain, have been considered as a standing and unchangeable punishment of the original disobedience of woman, and, by some physicians, as indispensably necessary to enable the uterus to relieve itself of its burden. By contemplating the numerous instances in which it has pleased God to bless the labours and ingenuity of man, in lessening or destroying the effect of the curse inflicted upon the earth, and by attending to the histories of the total exemption from pain in child-bearing that are recorded of the women in the Brazils, Calabria, and some parts in Africa, and of the small degrees of it which are felt by the Turkish women, who reduce their systems by frequent purges of sweet oil during pregnancy, I was induced to believe pain does not accompany child-bearing by an immutable decree of Heaven." (*Med. Inq. and Observations*, vol. iv. pp. 373, 374.)

Again:

"I have expressed a hope in another place, (Medical Repository, vol. vi.), that a medicine would be discovered that would suspend sensibility altogether, and leave irritability, or the powers of motion, unimpaired, and thereby destroy labour-pains altogether. I was encouraged to cherish this hope, by having known delivery to take place, in one instance, during a paroxysm of epilepsy, and having heard of another, during a fit of drunkenness, in a woman attended by Dr. Church, in both of which there was neither consciousness, nor recollection of pain." (Ib. p. 376.)

"Is it not singular to find," says Dr. Channing, "that in the city of our distinguished countryman, where was his fame, and where is his grave, that the great discovery of the age, the remedy of pain, should meet with the most uncompromising opposition? Is it not singular, that the power of fact, the origin and nutriment of all true philosophy, should not be felt and acknowledged in the very atmosphere of medicine and its collateral sciences? Upon those who sit in the seats of the prophets their mantle has fallen so gently, that it seems hardly to be felt. The shadow of the Apostle healed those by the way-side upon whom it fell. The shade of the past has come to us in its beauty and its power. It passes by us, and we hear its gentle, its noiseless word; but we have not faith, and shrink from its important teachings."

From this extract it will be seen, that our author lashes the townsmen of Rush with an unsparing hand, for what he conceives to be their uncompromising opposition to etherization—with what justice we do not exactly see. It is true that some have been less hasty to step into the waters when first troubled, than others, but, for ourselves, we have generally been willing to watch and profit by experience. The pages of the Medical Examiner have been freely open to both sides, and in the reference which is made to ourselves personally, by the author, more of opposition is accorded than we have ever asserted or claimed. We do not say this either in a recanting or complaining mood, but simply for explanation.

The volume is handsomely got up, and is, therefore, mechanically, as well as otherwise, worthy of a place in every good medical library.

