# Correction

**Published:** 1849-01

**Authors:** 


The editors of the Medical Examiner, take pleasure in giving insertion to the following from Dr. Huston:

Correction.—In the notice of Professor Channing's book on Etherization, contained in the last number of the Examiner, an error occurred in transcribing a paragraph from the work, which, as it does injustice to the author, the writer of the notice embraces the earliest opportunity of correcting it. It is in the following quotation: "It is no part of the purpose of the following treatise to teach, or to leave it to be inferred, that *no* untoward results have not followed, or will not again follow etherization." The word *no*, which we have put in italics, is not in the sentence in the book, and should not have appeared in the quotation.

